# Senior Co-Housing in the Netherlands: Benefits and Drawbacks for Its Residents

**DOI:** 10.3390/ijerph16193776

**Published:** 2019-10-08

**Authors:** Katja Rusinovic, Marianne van Bochove, Jolien van de Sande

**Affiliations:** 1 Faculty of Public Management, Law & Safety, The Hague University of Applied Sciences, 2521 EP The Hague, The Netherlands; 2 Erasmus School of Health Policy & Management, Erasmus University Rotterdam, 3000 DR Rotterdam, The Netherlands; vanbochove@eshpm.eur.nl (M.v.B.); vandesande@eshpm.eur.nl (J.v.d.S.)

**Keywords:** aging in place, senior co-housing communities, elderly care, loneliness, social support, informal care

## Abstract

Senior co-housing communities offer an in-between solution for older people who do not want to live in an institutional setting but prefer the company of their age peers. Residents of co-housing communities live in their own apartments but undertake activities together and support one another. This paper adds to the literature by scrutinizing the benefits and drawbacks of senior co-housing, with special focus on the forms and limits of social support and the implications for the experience of loneliness. Qualitative fieldwork was conducted in eight co-housing communities in the Netherlands, consisting of document analysis, interviews, focus groups, and observations. The research shows that co-housing communities offer social contacts, social control, and instrumental and emotional support. Residents set boundaries regarding the frequency and intensity of support. The provided support partly relieves residents’ adult children from caregiving duties but does not substitute formal and informal care. Due to their access to contacts and support, few residents experience social loneliness. Co-housing communities can potentially also alleviate emotional loneliness, but currently, this happens to a limited degree. The paper concludes with practical recommendations for enhancing the benefits and reducing the drawbacks of senior co-housing.

## 1. Introduction

In many countries, policies are directed towards enabling ‘aging in place’, which has led to a worldwide increase in the proportion of older people who remain part of their community [1]. Older people are now expected to stay at home longer, even in countries with long traditions of institutionalized care. Due to the closure of many residential care homes in the Netherlands, only people with severe care needs qualify for facility-based long-term care services [2].

It is widely accepted that older people are not only encouraged, but when possible also prefer to continue to live in their own house and neighborhood [1,3,4]. Older people are often strongly attached to their home, especially when they have lived in their house and community for a long time. Their home feels familiar, and that feeling enables a continuity of self and stimulates personal autonomy and individuality [3]. However, Bookman [5] (p. 423) argues, for many older people, aging in place in practice often leads to extreme isolation, as they have limited social contacts apart from ‘an occasional trip to the doctor or grocery store, or the occasional visit from a family member, friend, or home health aide’. A wide range of co-housing initiatives aim to offer an alternative form of aging in place, in between the extremes of living in an institutionalized setting and remaining in their own house. This form of housing is sometimes described as ‘living together on one’s own’ [6] (p. 44): Residents share common space and undertake activities together, without having to sacrifice their own dwelling and privacy. Co-housing initiatives exist in different forms, varying according to, among other things, the age and ethnicity of the residents, the presence of care facilities, the type of building, and the governance structure [5,6,7,8].

Previous research on co-housing initiatives has discussed background characteristics of people choosing this type of housing and their motives and experiences. Residents mainly report positive experiences, related to mutual assistance, solidarity, and a sense of community [7,8]. Although advantages usually get more attention, research also shows that co-housing can be challenging, particularly finding a balance between private life/individualism on the one hand and public life/collectivism on the other [7,8]. Furthermore, this type of housing might not be suitable for people with increasing care needs [7,9]. Labit [7], focusing on intergenerational co-housing, concluded that while ‘no one wants to leave their present accommodation’ (p. 42), residents said that in case of physical or mental deterioration, co-housing would no longer be suitable. They saw personal involvement and participation as prerequisites for being part of the community. Bernard et al. [9] found that residents with physical disabilities felt excluded by other residents, who associated physical disability with mental frailty. These findings suggest that senior co-housing communities do not necessarily protect residents from the experience of isolation and loneliness. The classic work of Hochschild [10] about an old age community showed that although living communities may seem egalitarian from the outside, in reality, a complex hierarchical system exists, ranging from honored residents at the top to less-respected ‘poor dears’ at the bottom (p. 58), cf. [11].

In this paper, we add to the literature by systematically investigating benefits and drawbacks of senior co-housing, with special focus on the forms and limits of social support and the implications for the experience of loneliness. We conceptualize social support in terms of instrumental and emotional support [12] and loneliness in terms of social and emotional loneliness [13] and examine their interrelations.

We conducted qualitative research in eight senior co-housing communities in the Netherlands. We selected The Hague as the main research site as the municipality of The Hague has a rich tradition when it comes to senior co-housing communities. With more than 60 co-housing communities, it is the largest ‘co-housing community city’ in the Netherlands [14]. Since the 1990s, the municipality of The Hague has tried to encourage community living in general and among seniors in specific. There is, for instance, an organization in The Hague which provides information and advice about co-housing, and in 2016, the municipality introduced subsidy to support and stimulate citizens of The Hague with the creation of a new co-housing community [15].

Based on document analysis, interviews, focus groups, and observations, we found that the benefits of co-housing are often balanced with corresponding drawbacks. The experience of a co-housing community as a small village means that residents look after one another, but it also means that there is gossip, and residents sometimes feel excluded. The instrumental and emotional support that residents offer one another partly relieves residents’ adult children from caregiving duties. However, residents set boundaries regarding the frequency and intensity of their support; their support does not substitute formal and informal care. Due to their access to contacts and support, few residents experience social loneliness, but co-housing is not a ready-made solution for emotional loneliness. The paper concludes with an overview of benefits and drawbacks, and recommendations for research and practice.

## 2. Materials and Methods

To obtain a closer understanding of co-housing communities and the perceptions of its residents, we conducted a qualitative study. Interviews with mostly open-ended questions allowed us to gain insight into the respondents’ experiences, beliefs, and actions. Following an adaptive theory approach, the aim of this study was not to generate a new theory from scratch but build on existing theories [16]. During the data collection and analysis, we used concepts from the literature on aging in place, informal care, and loneliness but also remained open to unexpected findings [17]. Below, we further explain different aspects of our research design.

The fieldwork started in October 2017 with interviewing key-informants (N = 17), in order to access senior co-housing communities. From March to June 2018, fieldwork was carried out in eight senior co-housing communities in the Netherlands.

A co-housing community is a type of housing inside a larger building that has housing as its main function and which consists of several housing units, whereby at least two households voluntarily share at least one living space, and, in addition, each have at least one private living space. As can be seen in Table 1, the majority of the residents we interviewed lived in co-housing communities in The Hague.

What the eight senior co-housing communities had in common was that they all were initiatives for low- and modest-income seniors (social housing) and had a common room where activities were organized. Furthermore, the first five initiatives were all registered as an association and had a residents’ association board. As such, they had their own regulations and had to organize at least two general residents’ meetings per year. The last three initiatives were recently established.

For this study, we interviewed a total of 32 seniors: Interviews relied on a semistructured questionnaire including questions about the residents’ motives for living in a co-housing community, advantages and disadvantages, social contacts, activities, social support, and loneliness. In addition, 6 (in)formal caregivers were interviewed about the care for residents of co-housing communities, using a topic list. Focus group sessions were also held with, among others, members of a residents’ association and students who lived with older residents (co-housing community H7). Furthermore, 5 hours of observations were conducted during 6 common activities organized by and for residents. Field notes were made during and after these informal conversations and observations. Finally, a document analysis of public records concerning the co-housing communities, such as the regulations, was conducted. The fieldwork was conducted by the authors and students from The Hague University of Applied Sciences and Erasmus University Rotterdam. In Table 2, an overview of the fieldwork is given.

In selecting our respondents, we aimed at a diverse research population with regard to gender, age, ethnic background, and care needs. The average age of the seniors we interviewed was 76 years. The youngest respondent was 60 and the oldest 93 years. The majority (two-thirds) of the respondents were women, and most were widowed or divorced. The vast majority of respondents had children (N = 28). Few of these children lived nearby. One-third of respondents were born outside the Netherlands. In selecting respondents, we tried to include both seniors who had been living in the collaborative housing initiative for some years, sometimes from the start, and relative newcomers. Three respondents had been living in the complex for more than 20 years, whereas four respondents had moved in the previous year.

All interviews were conducted face-to-face, recorded and transcribed verbatim. The interviews lasted on average an hour. One interview was conducted in English, another interview was conducted in Arabic, as these respondents spoke hardly any Dutch. An Arabic-speaking student acted as an interpreter during the interview. The other 30 interviews were conducted in Dutch. The interviews were analyzed with the software program Atlas-ti. We analyzed the data by adding codes such as loneliness, support, and the motivation to choose for a co-housing community. The codes were partially based on concepts from existing literature, such as emotional and social loneliness. Other codes were generated inductively, such as boundaries of support. The quotations were translated into English by the authors for the purpose of this article. When quotations are used, we use the housing initiative number (H1–H8, see Table 1) in combination with the respondents’ number, for example, I01-H1.

## 3. Benefits and Drawbacks of Senior Co-Housing Communities

We asked people living in communities what they experienced as advantages, limitations, and disadvantages of residing in a co-housing community, to discover whether such a community fits the idea of a supportive neighborhood and contributes to successful aging in place. Though respondents were predominantly positive about their experiences of residing in their community, it also became clear from our interviews and observations that not every older person would appreciate the communal aspect of a co-housing community. Below, we first present often mentioned benefits and drawbacks related to social contacts and social control. Then, we discuss the instrumental and emotional support respondents offer one another, and the boundaries thereof. Third, we answer the question what the available contacts and support mean for the experience of social and emotional loneliness.

### 3.1. Social Contacts and Social Control

The majority of respondents told us that the main advantage their co-housing community provided was the social contacts. For more than one third of the respondents, this was also the main reason they chose to live in a co-housing community. Respondents mentioned that it is relatively easy to get in contact with other residents, and as such, it is easier to ask their neighbors for help, have a chat, and undertake activities together than in general apartment blocks.

Because the (majority of the) residents were members of the same community, people knew each other and did not hesitate to ring each other’s doorbell. Residents stated that they were more than ‘just neighbors’. The respondents appreciated the sense of coziness and belonging the community offers, especially since their circle of friends and relatives had shrunk, they were not as physically mobile as they used to be, or they had anticipated their change in circumstances. A respondent described the value of having such coziness and a sense of belonging nearby:

*“The time will come when one of us will pass away. (..) Then of course you are not on your own. (..) If I were to live just somewhere in an apartment block, I would not seek company that easily. (..) Here it is already there.”*.

(I06-H2)

An extra enabling factor for this interaction is the communal room, where residents join activities such as coffee mornings, gymnastics, movie nights, drinks, cooking, and handicrafts. Respondents appreciated these activities, and some of them stated that they specifically chose co-housing because they wanted to stay active at their old age instead of sitting at home too often.

*“I thought ‘when I stop working, I do not want to end up being at home all day’. I want to stay active, be with other people and undertake things with them’. So that is why I chose the group thing.”*

(I03-H3)

*“The advantages are… the coziness, that is number one. Second is that once a month on Saturday we get together for a drink. (..) We just do that. With a snack, you know. And the opportunity that you can do many things. On Sunday afternoons there is a movie night and if you like to do yoga, you can join the yoga class on Thursdays.”*

(I04-H2)

The proximity of ‘good neighbors’ who look out for one another makes that people feel safe. Some respondents told us that as an older person, they felt vulnerable because they live on their own and are less physically mobile. Therefore, they appreciated the safe residing environment in the co-housing community. Residents know each other and do not open the central door of the building for strangers. People inform each other when they go on holidays and their son or daughter is taking care of the plants. Moreover, the structure of the building enables a clear view on who enters the building. These elements contribute to a sense of safety felt by residents.

*“I think that this co-housing community is definitely a good example when it comes to safety. (..) Safety is a plus for an older person. That you know that you don’t have to be afraid of anything.”*

(I03-H2)

Residents keep an eye on one another in a positive way. They make sure that everyone is doing well, for example. When someone does not show up for activities or is not seen for a while, residents ask one another if they have heard something or they try to get in touch with their fellow-resident on their own.

*“The advantage is that there is a certain amount of social control among us. I have this for example with X. If she hasn’t seen me for a week she will call me ‘hey Y, are you alright?”*

(I03-H2)

Respondents thus appreciated the social control that comes along with living in a co-housing community. Social control refers to the social norms and formal and informal rules and laws that are agreed upon. The social control provided them with social contacts and a feeling of safety by the relatively closed community. However, social control also has its downsides.

First of all, most of the co-housing communities in our case study are almost twenty years old. In this time, a closed system with certain written rules and unwritten norms has been created and instituted. Those rules and norms are, for example, about the use of the common room and the organization and attendance of activities, but also about more personal issues such as whether or not you are allowed to hang your laundry outside your house. Residents address each other’s inappropriate behavior when necessary. Some respondents disliked the paternalistic behavior taking place in their living community:

*“Of course, there have to be certain rules but I sometimes find them almost too strict. Then I think ‘that is a pity’. I understand that there have to be rules but for me it could be a bit more flexible. (..) I find it too rigid, too unyielding.”*

(I01-H3)

Second, the (in)formal rules and (un)written laws can lead to social exclusion and could pose a severe adjustment challenge for newcomers to the housing community. As a result, subgroups were present in several co-housing communities, cf. [10]. Those small groups often sit together, which negatively affects the group dynamic in the community:

*“There are people here who say ‘oh I am not going to sit next to him or her’. During our happy hour, this person sits over there and that person sits next to that one and that person sits next to that one. (..) One time there was a lady that wanted to sit somewhere. She told me that people looked at her saying ‘oh please, don’t take the seat next to mine’. She went home directly. Those things happen.”*

(I04-H1)

Third, respondents often compared the social norms within the community to ‘a small village’ where citizens know each other and keep an eye on each other, but also where gossip circulates and conflicts occur. Residents sometimes found those experiences annoying.

*“There is always gossip and rumor. That will always happen. (..) I am pleased that I regularly leave the building.”*

(I01-H1)

Some respondents told us about small irritations that eventually led to conflicts in their groups. These conflicts started, for example, as disagreements during general meetings or when the environment was noisy. Those conflicts often solved themselves but sometimes required mediation of the community board.

*“I clashed with one man during a meeting. Afterwards you sit together, talk about it and it is finished. You cannot prevent that something happens, that there is never a dispute or something. You just can’t.”*

(I02-H3)

The results show that a sense of community and social control are often seen as positive and are sometimes even idealized but can also lead to conflicts and disagreements within co-housing communities.

### 3.2. Offering Instrumental and Emotional Support to Co-Residents

Neighborly support is an important feature of senior co-housing communities. This is consistent with public policies throughout Europe, in which community-based care is increasingly promoted [18,19] and the need for informal and non-institutional support emphasized, such as that from family, friends, and neighbors [20,21,22]. However, this support has its boundaries. These boundaries are also evident within the co-housing communities. Residents agree that providing informal (health) care to other residents is not part of the deal. This view is in line with the regulations of various co-housing communities:

*“If a member is extremely ill or weakened to such an extent that daily care and/or nursing is required, then it cannot reasonably be expected that the other residents of the co-housing community will take on these tasks.”*

(Source: Regulations co-housing community H1)

In this section, we examine what kind of neighborly support residents give to other residents, and where residents draw the line between what types of support can and cannot be expected in co-housing communities. Following Van Dijk et al. [12], we distinguished instrumental and emotional support. Instrumental support refers to practical tasks such as going grocery shopping or picking up mail. However, it can also include household chores, helping with household finances, or transportation to the hospital. Emotional support involves having conversations and giving advice or comfort [12].

Offering instrumental and emotional support is very common within the co-housing communities. As stated in the previous section, residents mentioned that they were more than ‘just neighbors’. This means residents will help each other with all kinds of practical tasks, such as doing grocery shopping when someone is ill. Giving advice or having conversations with co-residents are also a matter of course. The following quotation stems from an interview with a resident who recently moved into a co-housing community:

*“I’ve already had several conversations with co-residents. People are often very open about what is going on and happening in their lives. There is someone in our co-housing community who lost his son. So, he told me that story. And that is of course quite emotional. But, you do share that with one another. You take care of one another if there is something going on.”*

(I01-H3)

However, in offering support, respondents often set boundaries. A respondent mentioned that he was always willing to drive someone to the hospital but would not attend the doctor’s appointment. Several respondents would see that as an invasion of the privacy of the person requesting the help and, moreover, considered it a task for family members. For the same reason, residents mentioned that they would not help someone with their administration or tax return.

*“I: And do you help other residents with their administration?*

*R: No, they don’t ask for that.*

*I: Because they don’t want that?*

*R: It’s a bit of a privacy issue, I think. If they have children, they ask the children. I always had someone who did the tax return and my son-in-law has been doing it for a few years now.”*

(I03-H4)

Another line is drawn when it comes to assisting with household chores. Some activities, such as doing grocery shopping for another resident, are taken for granted, especially when someone is ill.

*“R: If I have a neighbor and she happen to be sick, then I will offer to do the groceries. Yet, it was also made very clear to me by board members: ‘remember, you are not an informal caregiver’. That is certainly not the case, but you can help each other, that is the purpose of living together.*

*I: Looking after one another.*

*R: Yes, that you look after one another. And that really appeals to me. I like that.”*

(I01-H3)

Cleaning the house, on the other hand, only happens in exceptional cases. Residents explicitly do not want to become informal caregivers. Cleaning the house, as well as providing personal care (washing or showering) and medical assistance (for example, helping with medication), are seen as offering informal care to residents.

*“I: Where does informal care begin?*

*R: Well, if I have to put people in the shower. Real professionals should do that. But you know, that [community member] didn’t have a washing machine for some time, so I did her laundry.”*

(I01-H4)

As the above quote illustrates, exceptions were made. Where residents knew each other well, the limits of the types of support that could be given were stretched. Another important condition of emotional and instrumental support is that the support provided is temporary:

*“I: Where do you draw the line?*

*R: I think if it has to be done daily.*

*I: So it’s the frequency?*

*R: Yes.”*

(I01-H1)

The senior co-housing communities offer support to other residents that can relieve informal caregivers. One of the informal caregivers we interviewed said:

*“My mother is very happy in the co-housing community. I like the house, the neighborhood. And I think the advantage is that there are people nearby who know her. You actually have a circle of neighbors around you, good neighbors.”*

(IIC01-H6)

Nonetheless, that does not mean that the co-housing community can automatically cope with residents’ increasing care needs. In every co-housing community, there appeared to be elderly people who could no longer participate in activities because they were in poor health, cf. [9]. In earlier decades, these persons would have moved to a care home. Now, they continue to live at home and are only eligible for a nursing home level of care in case of severe care dependency.

*“A while ago, a community member was moved to a nursing home. But it was a drama before she left. She was affected with dementia and in the beginning you can still handle that, but after a while it got worse. And yes, at some point the situation just wasn’t tenable. She went out at night and then went to the beach and then she did not know where she lived. Or she went shopping or walking and was then brought home by strangers. And she often knocked at the door of her neighbor, also an older lady. That neighbor was completely stressed out.”*

(I01-H2)

The quotation illustrates that informal and formal caregivers are still indispensable for seniors living in co-housing community. At the same time, informal caregivers are alleviated to a certain extent by the instrumental and emotional support that is offered by residents in the co-housing community.

### 3.3. Loneliness in Co-Housing Communities

We examined whether social contacts and social support contribute to tackling a growing social and health concern: feelings of loneliness among older people. We asked the residents how they define loneliness and whether they ever feel lonely. Half of the respondents indicated that they felt lonely. This percentage is considerably lower than national statistics, which show that 74 percent of people aged 75 years or older feel lonely. If we look at marital status, it appears that respondents who live with a partner feel least lonely, whereas singles (widowers and divorced) feel most lonely [23].

The vast majority of respondents define loneliness as the lack of social contacts. One of the oldest respondents, a 92-year-old widow, said:

*“Of course, you can be alone, without feeling lonely. When I watch TV at night, I do not feel lonely. But I do feel lonely when I do not have anyone coming over on Sundays. I do not have anyone living nearby. Only a cousin in city A [in the eastern part of the Netherlands] and in city B [in the northern part of the Netherlands]. I don’t have anyone, except my son and daughter-in-law. But do I feel lonely? No, generally not. But you have to go after it yourself.”*

(I03-H1)

Social loneliness can be defined as the absence of an engaging social network, such as friends [13]. When asked why they do not feel lonely, respondents often mentioned living in a community, and especially the social contacts within the community. Some residents indicated that they experienced these social contacts in daytime as compensation for the hours they spent alone in the evening.

*“If you have spoken with enough people during the day, it is no problem for me to sit alone at home in the evening. I watch TV, or have the radio on.”*

(I01-H1)

Living in a community can contribute to diminishing social loneliness among the elderly. However, can co-housing also be a solution for *emotional* loneliness? Emotional loneliness can arise when a partner relationship ends through widowhood or divorce and is characterized by intense feelings of emptiness and abandonment [13]. In general, our findings suggest that co-housing communities cannot compensate for the loss of a partner, cf. [24]. To illustrate, the following quote comes from an interview with an 87-year-old widow:

*“I still miss my husband, especially when things happen with the children, for example. Then you realize that it is such a shame that you don’t have a husband anymore with whom you can talk about it.“*

(I04-H1)

Although she had been living in the same community for over 22 years, her emotional loneliness had not diminished. For her, feelings of loneliness are still strongly connected with the loss of her husband. Nevertheless, the emotional support given in co-housing communities can provide support after the death of a spouse:

*“If someone needs to clear his mind, or something bad has happened, you should be able to talk about it. For example, if a family member has died. Then the group will be there for you. We have already experienced this a few times.”*

(I02-H3)

In the old age community she studied, Hochschild [10] found that “the old among the old feel freer to talk about death” and are “unembarrassed” in their expression of grief (p. 87). However, some residents of the co-housing communities we studied mentioned that they keep their grief to themselves, instead of bothering others with it. In the terminology of Hochschild [10], they do not want to be categorized as ‘poor dears’ (p. 58). An 88-old widower, for instance, said:

*“After my wife passed away, I received quite some attention from fellow residents. Also, I was told several times: ‘let us know if you want to talk with someone.’ However, ... [long silence], you should not tell your story twice, because then you become annoying.”*

(I02-H2)

To conclude, loneliness is widely recognized as among the most significant and entrenched issues facing aging societies. Although co-housing communities are not a ready-made solution for loneliness, our findings suggest that co-housing contributes to combating feelings of social loneliness among older people. Emotional loneliness is part of the lives of many older people and is not solved by co-housing, but the emotional support that residents provide each other can alleviate it.

## 4. Discussion

This study found that senior co-housing communities offer various advantages to residents. In line with earlier studies, we found that older people living in co-housing communities value the available social contacts, social control, and social support [7,8]. In addition to previous research, this study paid special attention to the forms and limits of social support and the implications of co-housing for the experience of loneliness. We found that each of the benefits of co-housing was balanced by certain drawbacks (see Table 3 for an overview).

First of all, co-housing communities facilitate valuable social contacts between residents. Shared activities contribute to a sense of belonging. Residents have often known each other for a long time and are more than ‘just neighbors’. A drawback, however, is that for newcomers, it can be challenging to find their place within the community’s existing structure.

Second, and related to the first, many residents appreciate the social control that the co-housing community offers. The fact that residents look out for one another contributes to feeling safe and secure. However, in some cases, residents find the control too restrictive and paternalistic. A balance must be struck between collectivism and individualism [8].

Third, residents of co-housing communities offer one another instrumental and emotional support [12], but they do not want to be their neighbor’s informal caregiver. Residents are willing to do the groceries or take someone to a doctor’s appointment, but they do not want to provide personal care or forms of support that are perceived as ‘too personal’, such as help with financial administration. While the boundaries that residents draw are clear in practice, many of them actually do more; they make exceptions for residents who they think ‘deserve’ extra care and support. In other words, the ‘boundary work’ that these residents do depends on specific circumstances [22]. A benefit of supporting and, in some cases, caring for one another is that it contributes to easing the burden that many family caregivers experience in taking care of their parents [25]. However, for older people with severe care needs, senior co-housing has its limitations. Although they receive help from their neighbors and relatives, formal care services are still needed, and residents who need 24 h care cannot stay in the community. Co-housing communities thus appear to be inclusive for ‘deserving’ older people with moderate care needs but exclusive for ‘undeserving’ and/or highly care-dependent older people.

Lastly, but not least, the valuable contacts between residents and the support they provide one another can contribute to combating feelings of loneliness. Social loneliness, related to the absence of a support network, is uncommon among the residents of co-housing communities. However, co-housing is not a panacea for emotional loneliness. This type of loneliness can particularly arise when a partner relationship ends [13]. Many residents of co-housing communities, on losing their spouse and other loved ones, experience feelings of emptiness. Although the emotional support of fellow residents can alleviate emotional loneliness, some residents think they should not bother others with their feelings.

Our research has several implications for practice and future research. Senior co-housing has many benefits. The bottom–up character of many of these communities fits the idea of ‘active citizenship’ that is dominant in many welfare states today [26]. National and local governments, as well as housing associations, should not interfere too much with the establishment and functioning of co-housing initiatives but facilitate and support them where necessary. At the same time, senior co-housing communities should not be seen as a solution for all social, emotional, and physical problems that arise with an aging population. Senior co-housing communities do not provide a solution to severe care needs; formal care services are still needed for those who need much assistance. Co-housing communities in which care services are available for its residents [5] are a promising development. In reducing the drawbacks described above, the boards of co-housing communities can play an important role. By encouraging diversity based on age, gender, and ethnicity, communities can become more sustainable and inclusive. When it comes to residents’ feelings of emotional loneliness, co-housing communities could further help to alleviate feelings of emptiness by creating a safe environment in which residents feel free to share their feelings.

For future research, the governance structures and participation of older people in the development of new collective housing initiatives deserves attention. While current research often focuses on the micro-level practices and experiences of residents in co-housing communities, meso- and macro-level involvement of various public and private actors (such as municipalities, housing associations, no-profit organizations, elderly associations) is also important.

There are several limitations to this study. Although we studied multiple co-housing communities with diverse characteristics, our findings are not readily transferable or generalizable to other co-housing communities both in the Netherlands and in other national contexts. Moreover, although we strived for heterogeneity in the sample of residents, those who frequently participated in activities and who were community board members were overrepresented. Since our main focus was on the activities and experiences of residents, we only interviewed a limited number of formal and informal caregivers. A larger sample of informal caregivers could have added new insights into the question of whether and, if so, how co-housing communities relieve the burden of family caregivers.

## 5. Conclusions

Older people who live in a senior co-housing community experience both benefits and drawbacks associated with community life. The main benefit of such co-housing communities is the social contacts they provided. Residents stated that they were more than ‘just neighbors’. The co-housing community offers all kind of social activities, as well as emotional and instrumental support. It creates a sense of belonging among the residents. As such, living in a co-housing community can contribute to reducing social loneliness among its residents and contributes to feelings of social and personal safety. On the other hand, conflicts, disagreements, and processes of social exclusion are also part of community life. Moreover, co-housing alleviates emotional loneliness to a limited degree and is not suitable for people with severe care needs. National and local policymakers should further facilitate the potential of senior co-housing, without treating it as an alternative for care delivery.

## Figures and Tables

**Table 1:** Overview senior collaborative housing initiatives.

Housing Initiative	Year of Establishment	Residents’ Board	Healthcare Service Provided	Multi-Generation Home	City	Number of Respondents
H1	1994	Yes	No	No	The Hague	6
H2	2007	Yes	No	No	The Hague	6
H3	1997	Yes	No	No	The Hague	3
H4	1998	Yes	No	No	The Hague	5
H5	1998	Yes	No	No	The Hague	1
H6	2017	No	Yes	No	Rotterdam	2
H7	2012	No	Yes	Yes	Deventer	4
H8	2017	No	No	No	The Hague	5

**Table 2:** Overview of the fieldwork.

Fieldwork	N
Interviews with key informants	17
Interviews with residents	32
Interviews with (in)formal caregivers	6
Focus group interviews	2
Observations in common spaces	6

**Table 3:** Overview benefits and drawbacks of co-housing.

Benefits	Drawbacks
Social contacts and shared activities	Social exclusion; adjustment challenges for newcomers
Social control; looking out for one another	Limited freedom due to strict rules and norms; paternalism
Instrumental and emotional support; easing the burden of family caregivers	Boundaries based on frequency and intensity of support; distinctions between deserving/ undeserving residents
Diminishing social loneliness and alleviating emotional loneliness	Experience of boundaries in sharing feelings of emptiness
